# Sero-prevalence of hepatitis B and C viral infections in Ghanaian HIV positive cohort: a consideration for their health care

**DOI:** 10.1186/s12879-019-4027-y

**Published:** 2019-05-03

**Authors:** Faustina Pappoe, Charles Kofi Oheneba Hagan, Dorcas Obiri-Yeboah, Paul Nsiah

**Affiliations:** 1 Department of Microbiology and Immunology, School of Medical Sciences, College of Health and Allied Sciences, University of Cape Coast, Cape Coast, Ghana; 2 Department of Medical Biochemistry, School of Medical Sciences, College of Health and Allied Sciences, University of Cape Coast, Cape Coast, Ghana; 3 Department of Chemical Pathology, School of Medical Sciences, College of Health and Allied Sciences, University of Cape Coast, Cape Coast, Ghana

**Keywords:** HIV, HBV, HCV, Co-infection, Seroprevalence, Ghana

## Abstract

**Background:**

Antiretroviral therapy (ART) has significantly decreased HIV/AIDS-related morbidity and mortality. However, globally, many people living with HIV die from non-AIDS related illnesses including liver diseases which occur partly due to co-infection with HBV and or HCV. The aim of this study was to determine the seroprevalence of HBV and HCV among HIV infected individuals receiving care from three different hospitals in the Central Region of Ghana.

**Methods:**

This research was a case-case study. The population consisted of ART naive persons (newly confirmed HIV cases) and those who had been on ART for more than 3 months (old cases). Each individual’s sociodemographic characteristics and clinical data including their HBV and HCV status were collected. Those who knew their HBV and HCV status and those who did not know their status were tested for circulating HBsAg and anti-HCV using rapid diagnostic test cassettes. Descriptive analysis was done, and the data presented as median with interquartile range, frequency and percentage. Fisher’s exact test and Pearson Chi-square (χ^2^) test were used to determine associations between categorical variables.

**Results:**

Overall, 394 HIV individuals aged, 3 to 76 years old with a median age of 41 (IQR:34–49) participated in this study. Circulating HBsAg and anti-HCV were detected in 6.1% (24/394) and 0.5% (2/393) participants respectively with an overall seroprevalence of 6.6% (26/394). None of the participants was positive for both HBV and HCV infections. 92.1% (363/394) had no information on their HBV status while all the 394 participants did not know their HCV status during data collection. No significant association of HBV infection rate was found in all the socio-demographic data of the participants. But HBV infection rates were significantly higher in those at WHO clinical stages 2 and 3 (*P* = 0.004).

**Conclusion:**

HBV and HCV were detected among the HIV-infected participants. Majority of the participants had no information on their HBV status and none of the participants had information on his or her HCV status. This study recommends the need for policy makers to provide free HBV and HCV screening for all HIV infected individuals for their effective management.

## Background

The blood borne pathogens, Human Immunodeficiency Virus (HIV), Hepatitis B Virus (HBV), and Hepatitis C Virus (HCV) are still a significant worldwide public health challenge [1, 2]. Globally, it is estimated that almost 37 million people are infected or living with HIV and approximately two thirds of the infected individuals live in Sub-Saharan Africa [1]; more than 250 million and 70 million people are also estimated to be chronically infected with HBV and HCV, respectively [2]. In 2017, it was estimated that 940,000 deaths of the world’s HIV infected populations occurred due to AIDS-related illnesses [1]. The introduction of antiretroviral therapy (ART) has significantly decreased HIV/AIDS-related morbidity and mortality [3]. However, deaths resulting from non-AIDS-related illnesses have been on the increase. Worldwide, a large number of people living with HIV (PLHIV) die from non-AIDS illnesses including liver diseases [4]. The increased morbidity and mortality associated with liver diseases among PLHIV is partly due to co-infection with HBV and or HCV [2] and partly due to non-infectious agents. Thus, the global prevalence of HBV and HCV among PLHIV is 7.4 and 1.0% respectively [2].

Co-infection of HIV with viral hepatitis (HBV and/or HCV) occurs commonly because these viral infections share similar mode of transmission [5–7]. Both HBV and HCV are hepatotrophic viruses that account for 96% of all hepatitis mortality. They cause severe morbidity including cirrhosis and hepatocellular carcinoma (HCC) due to intra-hepatic apoptosis and mortality particularly among HIV-infected individuals [2, 8, 9]. The World Health Organization (WHO) recommends that all PLHIV including children regardless of their CD4 counts should initiate ART [10]. Those who are co-infected with chronic HBV irrespective of their alanine aminotransferase (ALT) level, hepatitis B e antigen (HBeAg) status or HBV DNA levels should have tenofovir disoproxil fumarate (TDF) in their ART regimen since it is active against HBV. Those co-infected with HCV should also be provided with direct-acting antiviral (DAA) drug which has been shown to have lesser drug-drug interactions with antiretroviral drugs compared to the older HCV regimen containing pegylated interferon and ribavirin [10, 11]. This treatment guideline is adopted in the Ghanaian setting where the patients are frequently monitored for liver fibrosis using aspartate aminotransferase (AST), alanine aminotransferase (ALT), aminotransferase/platelet ratio index (APRI) and all parameters of the liver function test every 6 months [12]. The purpose of this guideline is to help decrease liver-related morbidities and mortalities associated with HBV and or HCV co-infection [10]. Hence, knowledge on HBV and HCV status of PLHIV is vital for their effective management. However, in Ghana although HIV, HBV and HCV are public health issues, free HBV and HCV laboratory testing and treatment are not provided side-by-side HIV testing and treatment due to lack of resources and this might have negative effect on the efficacy of the regimens used to manage them. In Ghana, the estimated HIV prevalence is 1.47%, and that of HBV and HCV reported in the general population are 5.5–15.8% and 0.4–8.4% respectively [13–19]. However, studies on HBV and HCV among HIV infected individuals are limited and most of these studies were done at a single site, with HBV prevalence ranging from 2.4 to 41.7% while 1.0% has been reported for HCV [20, 21]. This study was a multi-centered one that aimed to determine the prevalence of HBV and HCV among HIV infected persons who were receiving care from three different hospitals in the Central Region of Ghana.

## Methods

### Study design, area and population

This study was a subset of a parent case-case study conducted on toxoplasmosis among HIV infected individuals from May to August 2015. The participants were recruited from three hospitals in the Central Region. The hospitals were the Cape Coast Teaching Hospital (CCTH) which is the referral and teaching hospital in the Central Region, Cape Coast Metropolitan Hospital (CCMH) both located in Cape Coast Metropolis and Saltpond Municipal Hospital (SMH) located in the Mfantseman District. All these hospitals provide services to HIV infected individuals within and beyond the Central Region. CCTH and SMH receive the highest number of infected individuals. CCTH is a tertiary facility with about 400 beds. The HIV/STIs clinic in the facility was the first to be established in the region in 2006 and has registered over 4000 HIV positive patients since. SMH has 112 beds and provides service to 517 HIV infected patients while CCMH has 120 beds with an estimated 365 HIV population.

### Sampling

A total of 394 HIV infected in-patients and out-patients of all age groups including children who were attending clinics at CCTH, CCMH and SMH in the Central Region were consecutively enrolled. The participants were of two sets: antiretroviral therapy (ART) naive individuals (newly confirmed HIV cases) and those who had been on ART for more than 3 months (old cases). The socio-demographic characteristics, clinical data including their WHO HIV clinical stage 1, 2, 3 or 4 and comorbid conditions including their HBV and HCV status were collected. The data were obtained from the patients or guardians through structured and standardized questionnaire interview, medical examination records, and informants including physician or medical personnel. Venous blood samples (2-5 mL) were drawn into sterile sodium citrate tubes and centrifuged at 2500 x g for 10 min. The plasma was collected in Eppendorf tubes and stored at − 80 °C until tested. Those who knew their HBV and HCV status and those who did not know their status were both tested.

### Serology

Screening of the participants for circulating hepatitis B surface antigen (HBsAg) and anti-HCV was done using rapid diagnostic test cassettes (Innovita Biological Technology Co. Ltd., Beijing China). Testing was done following the manufacturer’s protocol. Briefly, a drop of the plasma was transferred into the sample well (S) and the result was read within 10 to 15 min. The result was considered positive when two purple bands appeared at both the control line (C) and the test line (T), and negative when only one colour band was observed at the control line (C). The sensitivity and specificity for the test kits were 98 and > 99% respectively for both HBsAg and anti-HCV test kits.

### Statistical analysis

The collected data were analyzed using SPSS version 16.0 (Chicago, SPSS Inc.). Descriptive analysis was done to determine median with interquartile range, frequency and percentage of the variables of the participants. Fisher’s exact test and Pearson Chi-square were used to assess the differences in the proportions of HBsAg positivity between categorical variables. *P* value ≤0.05 was considered statistically significant.

## Results

### The sociodemographic and clinical data of the participants

For the 394 HIV-infected patients who participated in this study, majority was recruited from CCTH 217(55.1%) followed by SMH 149(37.8%) with the lowest proportion 28(7.1%) obtained from CCMH (Table 1). In total, there was a wide range of ages among the participants, namely 3 to 76 years old with median age of 41 years with IQR of 49–34 years and there were fewer males than females, thus, 96(24.37%) males and 298(75.63%) females. In terms of educational status, most of the participants had attained basic education [257(65.2%)], few had attained secondary education [42(10.7%)] and fewer had attained tertiary education [18(5.6%)] while close to fifth few had never been to school [77(19.5%)]. Occupationally, majority of the participants were unemployed [300(76.1%)]; few were employed in the informal sector of the economy [63(16.0%)] and less than a tenth were employed in the formal sector [31(8%)]. The marital status of the participants was varied, ranging from married/cohabiting [218(55.3%)], single [63(16.0%)], divorced [60(15.2%)] to widow/widower [53(13.5%)] in decreasing order of magnitude. Those who were residents of rural areas constituted the highest proportion [266(67.5%)].

**Table 1:** Socio-demographic characteristics of the participants enrolled in this study

Variables	CCTH *n* = 217 (55.1%)	CCMH *n* = 28 (7.1%)	SMH *n* = 149 (37.8%)	Total N (%)
Age (years)				
≤ 19	6 (2.8)	1 (3.6)	5 (3.4)	12 (3.1)
20–29	21 (9.7)	8 (28.6)	15 (10.1)	44 (11.2)
30–39	61 (28.1)	6 (21.4)	51 (34.2)	118 (30.0)
40–49	76 (35.0)	8 (28.6)	41 (27.5)	125 (31.7)
50–59	42 (19.4)	4 (14.3)	25 (16.8)	71 (18.0)
≥ 60	11 (5.1)	1 (3.6)	12 (8.1)	24 (6.1)
Median (IQR)	42 (34.5–49)	36 (26.7–543)	41 (35–49.5)	41 (34–49)
Gender				
Male	49 (22.6)	4 (14.3)	43 (28.9)	96 (24.4)
Female	168 (77.4)	24 (85.7)	106 (71.1)	298 (75.6)
Educational status				
None	46 (21.2)	5 (17.9)	26 (17.4)	77 (19.5)
Basic	136 (62.7)	17 (60.7)	104 (69.8)	257 (65.2)
Secondary	23 (10.6)	6 (21.4)	13 (8.7)	42 (10.7)
Tertiary	12 (5.5)	0 (0.0)	6 (4.0)	18 (5.6)
Employment				
Unemployed	35 (16.1)	5 (17.9)	23 (15.4)	63 (16.0)
informal	163 (75.1)	22 (78.6)	115 (77.2)	300 (76.1)
formal	19 (8.8)	1 (3.6)	11 (7.4)	31 (7.9)
Marital status				
Single	36 (16.6)	6 (21.4)	21 (14.1)	63 (16.0)
Married/Cohabiting	121 (55.8)	19 (67.9)	78 (52.4)	218 (55.3)
Widow/widower	32 (14.8)	1 (3.6)	20 (13.4)	53 (13.5)
Divorced	28 (12.9)	2 (7.1)	30 (20.1)	60 (15.2)
Residence				
Rural	147 (67.7)	19 (67.9)	100 (67.1)	266 (67.5)
Urban	70 (32.3)	9 (32.1)	49 (32.9)	128 (32.5)

With regards to clinical data, of the 394 participants, approximately 47(12%) were newly infected HIV ART naïve individuals while the rest [347(88.1%)] were old cases (Table 2). They included 271(68.8%) WHO stage 1, 78(19.8%) stage 2, 29(7.4%) stage 3 and 16(4.1%) stage 4 infected individuals. Nevertheless, extremely high proportion 363(92.1%) of the participants had no information on their HBV status coupled with the fact that none of the participants had information on his/her HCV status when the data were collected.

**Table 2:** Clinical characteristics of the participants

Variables	CCTH *n* = 217 (55.1%)	CCMH *n* = 28 (7.1%)	SMH *n* = 149 (37.8%)	Total N (%)
HIV patients				
Newly confirmed cases	29 (13.4)	7 (25.0)	11 (7.4)	47 (11.9)
Old cases	188 (86.6)	21 (75.0)	138 (92.6)	347 (88.1)
WHO clinical stage				
Stage 1	137 (63.1)	20((71.4)	114 (76.5)	271 (68.8)
Stage 2	52 (24.0)	4 (14.3)	22 (14.8)	78 (19.8)
Stage 3	21 (9.7)	1 (3.6)	7 (4.7)	29 (7.4)
Stage 4	7 (3.2)	3 (10.7)	6 (4.0)	16 (4.1)
HBV/HCV status before testing				
HBV known	19 [4*] (21.1)	0 (0)	12(*P* = 1; 8.3)	31 (7.9)
HBV unknown	198 (91.2)	28 (100)	137 (91.9)	363 (92.1)
HCV unknown	217 (100)	28 (100)	149 (100)	394 (100)

P = [*] = number of positive case(s)

### HBV and HCV seroprevalence among the participants

Circulating HBsAg and anti- HCV were detected in 24 and 2 respectively of the 394 participants with an overall seroprevalence of 26/394(6.6%) (Table 3). None of the participants was positive for both HBV and HCV infections (Table 3). The 24 HBV positive individuals included 5/24(21%) already known HBV positive patients who also tested positive when tested again in this study while the remaining 19/24(79.2%) were part of those who had no information on their HBV status (92.1%; Table 2). With regards to seroprevalence in relation to the patient’s data, HBV was detected in nearly all the age groups. The lowest 1(2.3%) and highest 11(9.3%) positive rate were detected in the participants within the age groups 20–29 and 30-39 years respectively. Infection in females [19(6.4%)] was higher than in males [5(5.2%)]. Moreover, the positive rate was higher among those who had never been to school [5(6.5%)] and those who had attained basic education [17(6.6%)]. Approximately 2(5%) of those who had attained secondary education was positive for HBsAg whereas no infection was detected among those with tertiary education background (Table 3). Also, 20 (6.7%) was observed among those with informal jobs which was higher than that in those who were unemployed [3(4.7%)] and those with formal employment [1(3.2%)]. In relation to the marital status, divorced participants had the highest positive rate [5(8.3%)] followed by married/cohabiting [16(7.3%)], single [2(3.2%)] and the widow/widower [1(1.9%)]. Those who were living in rural areas had a slightly higher positive rate [17(6.4%)] compared to those in the urban areas [7(5.5%)]. The differences in the positive rates within each of the variables under investigation in this study were observed not to be statistically significant (Table 3).

In addition, the positive rate of HBV was higher among the HIV old cases [23(6.6%)] than the newly diagnosed cases [1(2.1%)] but this difference was not significant (*P* = 0.376). Also, slightly more than 12(15%) of WHO stage 2 patients were infected with HBV followed by stage 3 [2(6.9%)], stage 1 [10(3.7%)] and finally none of the stage 4 patients was infected. This difference was found to be statistically significant (*P* = 0.004).

The individuals [2 (0.5%)] who were positive to anti-HCV belonged to age groups 30–39 and 40–49 years and were all females (Table 3). HCV infection was detected only among those with secondary education [1(2.4%)] and those who had never been to school [1(1.3%)]. Also, the infected persons were among those who were unemployed [1(1.6%)] and had informal employment [1(0.3%)], married/cohabiting [2(0.9%)], living in rural areas [2(0.8%)], belonged to HIV old case group [2(0.6%)] and WHO clinical stages 2 [1(1.3%)] and 4 [1(6.3%)]. No statistical tests for association of infection rates in the groups was assessed due to the limited number of positivity.

**Table 3:** The demographic and clinical characteristics of the participants in relation to HBV and HCV infection

Variables	N (%)	Seroprevalence of HBV coinfection (%)	*P* value	Seroprevalence of HCV coinfection (%)
Age (years)				
≤ 19	12 (3.1)	0 (0.0)	0.464	0 (0.0)
20–29	44 (11.2)	1 (2.3)		0 (0.0)
30–39	118 (30.0)	11 (9.3)		1 (0.9)
40–49	125 (31.7)	8 (6.4)		1 (0.8)
50–59	71 (18.0)	3 (4.2)		0 (0.0)
≥ 60	24 (6.1)	1((4.2)		0 (0.0)
Gender				
Male	96 (24.4)	5 (5.2)	0.809	0 (0.0)
Female	298 (75.6)	19 (6.4)		2 (0.7)
Educational status				
None	77 (19.5)	5 (6.5)	0.903	1 (1.3)
Basic	257 (65.2)	17 (6.6)		0 (0.0)
Secondary	42 (10.7)	2 (4.8)		1 (2.4)
Tertiary	18 (5.6)	0 (0.0)		0 (0.0)
Employment				
Unemployed	63 (16.0)	3 (4.7)	0.188	1 (1.6)
Informal	300 (76.1)	20 (6.7)		1 (0.3)
Formal	31 (7.9)	1 (3.2)		0 (0.0)
Marital status				
Single	63 (16.0)	2 (3.2)	0.240	0 (0.0)
Married/Cohabiting	218 (55.3)	16 (7.3)		2 (0.9)
Widow/widower	53 (13.5)	1 (1.9)		0 (0.0)
Divorced	60 (15.2)	5 (8.3)		0 (0.0)
Residence				
Rural	266 (67.5)	17 (6.4)	1.000	2 (0.8)
Urban	128 (32.5)	7 (5.5)		0 (0.0)
HIV patients				
Newly confirmed cases	47 (11.9)	1 (2.1)	0.376	0 (0.0)
Old cases	347 (88.1)	23 (6.6)		2 (0.6)
WHO clinical stage				
Stage 1	271 (68.8)	10 (3.7)	**0.004**	0 (0.0)
Stage 2	78 (19.8)	12 (15.4)		1 (1.3)
Stage 3	29 (7.4)	2 (6.9)		0 (0.0)
Stage 4	16 (4.1)	0 (0.0)		1 (6.3)
HBV/HCV status after testing in this study				
HBV	394	19/394 (5. 3)^a^; 5/394 (1.27)^b^		
HCV	394 (100)			2 (0.5)
Total hepatitis seroprevalence	26 (6.6)	24 (6.1)		2 (0.5)

^a^ = new HBV positive case detected in this study

^b^ = Old HBV positive case who tested positive too when tested again in this study

*P* value in bold indicates variable with evidence of association

## Discussion

People living with HIV have a greater risk of being co-infected with HBV and or HCV compared to the general population because these infections share a common mode of acquisition [5, 15–18, 20]. In Ghana, data on investigating simultaneously the prevalence of both HIV-HBV and HIV-HCV co-infection remain limited. This study aimed at determining the prevalence of HBV and or HCV co-infection among HIV infected persons from three different hospitals in the Central Region of Ghana. This study reports an overall viral hepatitis seroprevalence of 6.6% (26/394): Twenty-four or 6.1% of the participants were HIV-HBV co-infected (i.e. had current infection) while only 2 or 0.5% were HIV-HCV co-infected (i.e. had current/past infection) with no person co-infected with both hepatitis viruses. The prevalence of HIV-HBV co-infection in this study is slightly low compared to most previous reports on HBV-HIV co-infection from other regions of Ghana between 1999 and 2016 (8.3–41.7%) [20] (with the exception of one study that reported lower seroprevalence of 2.4% [20]) and other studies from other sub-Sahara countries such as Sudan (11.7%) [22]; Nigeria (7.9%) [23] and Uganda (16.9%) [24]. Similarly, HCV prevalence in this study is lower than a previous study in Ghana and reports from elsewhere ranging from 1 to 43.4% [21–25]. These HBV and or HCV seroprevalence patterns are not different from previous studies from Ghana that involved the general population (i.e. HIV uninfected individuals) where the seroprevalences in most cases ranged from 6.94 to 15.8% for HBsAg and 1.84 to 8.4% for HCV [14–17, 19]. Only two studies reported lower seroprevalences, 5.5% for HBsAg [18] and 0.4% for HCV [16]. Comparing the HBV and HCV results of this current study to previous reports of studies conducted in Ghana and other sub-Sahara African countries [22–24, 26] it could be observed that HBV seroprevalence is higher than HCV seroprevalence in the Ghanaian population and the sub-Sahara African countries. Thus, it is important to screen all HIV infected individuals in sub-Sahara countries for HBsAg prior to ART.

To the best of our knowledge based on literature review, this is the first study to evaluate the HBV and HCV status of the PLHIV in the Central Region of Ghana. It was found that majority (92.1%) of the participants had no information on their HIV-HBV co-infection status and this was not different from HIV-HCV status where none of the 394 participants knew his/her status. This finding confirms that many PLHIV in most developing countries particularly sub-Sahara African countries do not know their co-infection status [27]. This might be due to the fact that the test is not free of charge. Hence testing is optional for the individuals and this may have a negative effect on their life expectancy since HBV and HCV are associated with serious complications such as cirrhosis and hepatocellular carcinoma which in turn increases mortality among PLHIV.

Additionally, we could not perform statistical tests for association for the HCV positivity and the variables because very few, two persons were co-infected with HCV. Statistical analysis of HIV- HBV co-infection did not show significant association in the HBV infection rate in all the socio-demographic data of the participants (in age groups, gender, educational status, employment, marital status and residence). This result is partly consistent with the findings of Sagoe et al. (2012), who reported that HBV infection among HIV infected persons recruited from Greater Accra Region of Ghana was not associated with age or gender [28]. Kye-Duodu et al. (2016) also found no significant association in HBV infection among PLHIV from rural and urban areas in the Eastern Region of Ghana but in the same study, HBV infection was found to be significantly higher in adults (≥18 years). In the Ghanaian general population, HBV infection was reported to be significantly higher in men than women [16] which is contrary to the result of this study. A study from Rwanda also found discordant relationship where older age was associated with HBV infection, but gender was not associated with infection [29]. Our finding also differs from a report from Cameroon where HBsAg positivity was found to be higher among those living in urban areas than those in the rural areas [30]. Outside sub-Sahara Africa, this result on HBV is in accordance with the findings reported in Nepal [31]. These discrepancies could be due to several factors like differences in the population, geographical areas and differences in infrastructure.

Nevertheless, significant association was seen in the HBV positive rate among WHO clinical stages. Generally, the infection rates were significantly higher in participants at WHO clinical stages 2 and 3. From the literatures reviewed no study from Ghana was found to have reported a result relating to this aspect of our study. Rusine et al. (2013) from Rwanda reported similar finding where HBV infection was also found to be significantly higher in patients at WHO clinical stages 3 or 4 compared to stage 1 [29]. This study also showed no significant association of HBV positivity in old HIV and new HIV infected patients. The major limitation of this study was that the viral DNA and markers of liver-related diseases such as aspartate aminotransferase (AST) and alanine aminotransferase (ALT) could not be determined as well as the CD4 counts of the patients at the various WHO clinical stages due to limited resources.

## Conclusions

HBV and HCV were detected among the HIV-infected participants. Extremely high proportion had no information on their HBV status, and none had any information on his or her co-infection status before screening in this study. This study recommends the need for policy makers to provide free HBV and HCV screening for all HIV infected individuals for clinician to provide effective management to them including optimal selection of treatment especially for newly diagnosed HIV patients and also in case of switching an individual from first-line ART to second-line ART due to treatment failure. The authors further recommend that the possibility of HBV infected persons rapidly progressing to AIDs stage be investigated.

## Abbreviations

AIDS: Acquired Immunodeficiency syndrome
ALT: Alanine aminotransferase
APRI: Aminotransferase/platelet ratio index
ART: Antiretroviral therapy
AST: Aspartate aminotransferase
CCMH: Cape Coast Metropolitan Hospital
CCTH: Cape Coast Teaching Hospital
CD4: Cluster of Differentiation
DNA: Deoxyribonucleic acid
HBeAg: Hepatitis B e antigen
HBsAg: Hepatitis B surface antigen
HBV: Hepatitis B virus
HCV: Hepatitis C virus
HIV: Human Immunodeficiency Virus
PLHIV: People Living with HIV
SMH: Saltpond Municipal Hospital
STIs: Sexually transmitted infections
TDF: Tenofovir disoproxil fumarate
UCC: University of Cape Coast
WHO: World Health Organization

## References

[1] UNAIDS, 2017 Global HIV statistics. 2018: Fact sheets. http://www.unaids.org/sites/default/files/media_asset/UNAIDS_FactSheet_en.pdf. Accessed 12 Aug 2018.

[2] WHO. Global hepatitis report (2017). Licence: cc by -NC-SA 3.0IGO.

[3] Obiri-Yeboah D (2018). Immunologic and virological response to ART among HIV infected individuals at a tertiary hospital in Ghana. BMC Infect Dis.

[4] Farahani M (2017). Prevalence and distribution of non-AIDS causes of death among HIV-infected individuals receiving antiretroviral therapy: a systematic review and meta-analysis. Int J STD AIDS.

[5] Ferreira-Junior ODC (2018). Prevalence estimates of HIV, syphilis, hepatitis B and C among female sex workers (FSW) in Brazil, 2016. Medicine (Baltimore).

[6] Alter MJ (2006). Epidemiology of viral hepatitis and HIV co-infection. J Hepatol.

[7] Platt L (2016). Prevalence and burden of HCV co-infection in people living with HIV: a global systematic review and meta-analysis. Lancet Infect Dis.

[8] Dervisevic S, Pillay D (2003). Issues in diagnostic testing and monitoring in HIV/viral hepatitis co-infection. J HIV Ther.

[9] Chun HM (2012). Hepatitis B virus coinfection negatively impacts HIV outcomes in HIV seroconverters. J Infect Dis.

[10] WHO (2016). Consolidated guidelines on the use of antiretroviral drugs for treating and preventing Hiv infection.

[11] WHO. Guidelines for the screening, care and treatment of persons with chronic hepatitis C infection. Geneva; 2016. 27227200

[12] Stockdale AJ (2015). Liver fibrosis by transient Elastography and Virologic outcomes after introduction of Tenofovir in lamivudine-experienced adults with HIV and hepatitis B virus coinfection in Ghana. Clin Infect Dis.

[13] Commission GA (2016). Ghana national HIV and AIDS strategic plan 2016-2020.

[14] Martinson FE (1996). Seroepidemiological survey of hepatitis B and C virus infections in Ghanaian children. J Med Virol.

[15] Ampofo W (2002). Prevalence of blood-borne infectious diseases in blood donors in Ghana. J Clin Microbiol.

[16] Yakass MB (2016). Prevalence of blood borne viruses in IVF: an audit of a fertility Centre. JBRA Assist Reprod.

[17] Ephraim R (2015). Seroprevalence and risk factors of hepatitis B and hepatitis C infections among pregnant women in the Asante Akim north municipality of the Ashanti region, Ghana; a cross sectional study. Afr Health Sci.

[18] Ephraim R (2014). Seroprevalence of hepatitis B and C viral infections among type 2 diabetics: a cross-sectional study in the Cape Coast Metropolis. Ann Med Health Sci Res.

[19] Lokpo SY (2017). Viral hepatitis Endemicity and trends among an asymptomatic adult population in ho: a 5-year retrospective study at the ho municipal hospital, Ghana. Hepat Res Treat.

[20] Agyeman AA, Ofori-Asenso R (2016). Prevalence of HIV and hepatitis B coinfection in Ghana: a systematic review and meta-analysis. AIDS Res Ther.

[21] King S (2015). Antibody screening tests variably overestimate the prevalence of hepatitis C virus infection among HIV-infected adults in Ghana. J Viral Hepat.

[22] Mudawi H (2014). Overt and occult hepatitis B virus infection in adult Sudanese HIV patients. Int J Infect Dis.

[23] Tremeau-Bravard A (2012). Seroprevalence of hepatitis B and C infection among the HIV-positive population in Abuja, Nigeria. Afr Health Sci.

[24] Baseke J, Musenero M, Mayanja-Kizza H (2015). Prevalence of hepatitis B and C and relationship to liver damage in HIV infected patients attending joint clinical research Centre clinic (JCRC), Kampala, Uganda. Afr Health Sci.

[25] Li CW (2018). Changing seroprevalence of hepatitis C virus infection among HIV-positive patients in Taiwan. PLoS One.

[26] Barth RE (2010). Hepatitis B/C and HIV in sub-Saharan Africa: an association between highly prevalent infectious diseases. A systematic review and meta-analysis. Int J Infect Dis.

[27] Coffie PA (2017). Trends in hepatitis B virus testing practices and management in HIV clinics across sub-Saharan Africa. BMC Infect Dis.

[28] Sagoe KW (2012). Prevalence and impact of hepatitis B and C virus co-infections in antiretroviral treatment naive patients with HIV infection at a major treatment center in Ghana. J Med Virol.

[29] Rusine J (2013). High seroprevalence of HBV and HCV infection in HIV-infected adults in Kigali, Rwanda. PLoS One.

[30] Noubiap JJ (2015). Hepatitis B and C co-infections in some HIV-positive populations in Cameroon, west Central Africa: analysis of samples collected over more than a decade. PLoS One.

[31] Bhattarai M (2018). Epidemiological profile and risk factors for acquiring HBV and/or HCV in HIV-infected population groups in Nepal. Biomed Res Int.

